# Heat and mass flux analysis of magneto-free-convection flow of Oldroyd-B fluid through porous layered inclined plate

**DOI:** 10.1038/s41598-022-27265-w

**Published:** 2023-01-12

**Authors:** Muhammad Bilal Riaz, Aziz Ur Rehman, Adam Wojciechowski, Abdon Atangana

**Affiliations:** 1 Department of Mathematics, University of Management and Technology, Lahore, 54770 Pakistan; 2 Faculty of Technical Physics, Information Technology and Applied Mathematics, Lodz University of Technology, 90-924 Lodz, Poland; 3 Institute for Groundwater Studies (IGS), University of the Free State, Bloemfontein, 9301 South Africa

**Keywords:** Engineering, Mathematics and computing

## Abstract

The present work examines the analytical solutions of the double duffusive magneto free convective flow of Oldroyd-B fluid model of an inclined plate saturated in a porous media, either fixed or moving oscillated with existence of slanted externally magnetic field. The phenomenon has been expressed in terms of partial differential equations, then transformed the governing equations in non-dimensional form. On the fluid velocity, the influence of different angles that plate make with vertical is studied as well as slanted angles of the electro magnetic lines with the porous layered inclined plate are also discussed, associated with thermal conductivity and constant concentration. For seeking exact solutions in terms of special functions namely Mittag–Leffler functions, G-function etc., for Oldroyd-B fluid velocity, concentration and Oldroyd-B fluid temperature, Laplace integral transformation method is used to solve the non-dimensional model. The contribution of different velocity components are considered as thermal, mass and mechanical, and analyse the impacts of these components on the fluid dynamics. For several physical significance of various fluidic parameters on Oldroyd-B fluid velocity, concentration and Oldroyd-B fluid temperature distributions are demonstrated through various graphs. Furthermore, for being validated the acquired solutions, some limiting models such as Newtonian fluid in the absence of different fluidic parameters. Moreover, the graphical representations of the analytical solutions illustrated the main results of the present work and studied various cases regarding the movement of plate.

## Introduction

The process of heat and mass transfer has a great importance from the industrial point of view. Many researchers and scientists concentrate on this area. In modern technologies and various industrial fields, the non-Newtonian fluid theory has extensive impact because Newtonian fluid model can not express many flow characteristics. A non-Newtonian fluid that obeys the nonlinear relationships between the rate of shear strain and the shear stress. The non-Newtonian fluid theory has significant utilization in modern engineering, especially in petroleum industry used to extract crudeoil from different petroleum productions. The properties of Newtonian fluid in most of the cases are not valid but scientists desire to model the complex models for non-Newtonian fluid. The importance of non-Newtonian fluid has been enlarged from the last few decades, specifically in the research field. The non-Newtonian fluids have numerous ever-increasing applications in industrial sectors, but some specific are mentioned here, such as at large-scales reducing and enhancing heating/cooling systems, biochemical and process engineering, extrusion of molten plastic in industry, reducing oil pipe line friction, polymer processing, reducing fluid friction, well drilling, flow tracers, biological materials, biomedical flow analysis, plastic foam processing, lubrication processes, food processing industries, chemical processing, all emulsions, handling of muds, slurries and complex mixtures. Many researchers and scientists focused on non-Newtonian fluid while considering different fluid geometries. Therefore, simulating and modelling the flow phenomena of non-Newtonian fluid that is facilitated and play the important role in human life. Researchers investigated different non-Newtonian fluid models regarding physical and computational characteristics such as second grade model, viscoplastic model, power law model, Bingham plastic model, Jeffery model, Oldroyd-B fluid model, Brinkman type model, Casson model, Walters-B fluid model and Maxwell model^1–7^, that different fluid models exists in the literature have various characterictics or certain limitations,for instance, second grade fluid model efficiently explained the elasticity but does not discuss the viscosity, the power law model described the features of viscosity but failed to explain the impacts of elasticity, which motivate/attract the researchers and mathematicians towards the study of such complex fluids. Systematic analysis of such fluid flow models have significantly important for theoretical studies and practical implementations in modernistic mechanization. Among several proposed mathematical models for such fluids (non-Newtonian), Maxwell fluid attracted special attention because of its simplicity, also that can be predicted stress relaxation, which is the commonest non-Newtonian fluid due to its more extensive applications and substantial role in different fields serving as mechanical as well as chemical applications, bio engineering operations, metallurgy and especially in food processing industries. In 1867, James Clerk was first proposed the Maxwell fluid model, and James G. Oldroyd popularized the idea, a few years later^8,9^, with an aim to predict the visco elastic behavior of air^10^. Multiple products such as honey, soup, jelly, china clay, tomato sauce, artificial fibers, synthetic lubricants, concentrated fruit juices, pharmaceutical chemicals, paints and coal, etc. are some applied illustrations of such fluid. The study of Oldroyd-B fluid movement in the context of fluid mechanics, was explored by several mathematicians, scientists, researchers and engineers that depends upon various situations because of its naturalness. Some interesting features regarding different fluid models on flow, mass and energy transfer characteristics are mentioned in^11,12^.

Herbert et al.^13^ provided a review on essential advances in double diffusive convection. Hughes et al.^14^ examined double diffusive convection taking two stabilizing gradients into account and analyzed the weird results of magnetic buoyancy. Ehrenstein et al.^15^ studied a Chebyshev collocation method aimed at Navier–Stokes equations considering application to double-diffusive convection. Srinivasacharya et al.^16^ investigated double diffusive natural convection with respect to power-law fluid saturated porous medium under Soret and Dufour Effects. Asha et al.^17^ examined double diffusion on peristaltic flow of nanofluid under the influences of magnetic field, porous medium, and thermal radiation. Krishna et al.^18^ analysed the hall and ion slip effects on unsteady MHD free convective rotating flow through a saturated porous medium over an exponential accelerated plate. Hall and ion slip effects on MHD rotating boundary layer flow of nanofluid past an infinite vertical plate embedded in a porous medium are studied by Krishna et al.^19^. Chamkha^20^ investegated the non-Darcy fully developed mixed convection in a porous medium channel under the influence of heat generation/absorption and hydromagnetic effects. Heat and mass transfer analysis of unsteady hybrid nanofluid flow over a stretching sheet with thermal radiation is examined by Sreedevi et al.^21^. Chamkha et al.^22^ discussed the effect of heat generation or absorption on thermophoretic free convection boundary layer from a vertical flat plate embedded in a porous medium. Murthy et al.^23^ examined the heat and mass transfer in a two-dimensional magnetohydrodynamic free convection boundary layer flow along a vertical semi-infinite flat surface immersed in a thermal and mass stratified Darcy porous medium under the influence of the Soret and Dufour effect. Two cases for MHD free convection flow are investigated by Narahari and Debnath^24^ such as: magnetic field is fixed regarding fluid (MFFRF) and other case is magnetic field is fixed regarding plate (MFFRP). Later on, both cases for MFFRF and MFFRP along with a chemical reaction and varying wall temperature, the results are extended by Shah et al.^25^. The parabolic partial differential equations governing the flow in the presence of a magnetic field, Hall currents and the free stream velocity has been studied by Takhar et al.^26^. Modather et al.^27^ presented the analytical solution of the problem related to MHD mass and heat transfer of an oscillatory two-dimensional viscous fluid that is electrically conducting over an infinite vertical permeable moving plate which is embedded in a porous medium along with a chemical reaction and transverse magnetic field. Prabhakar et al.^28^ investigated the impact of viscous dissipation numerically on MHD fluid flow over an oscillating vertical plate that is embedded in porous medium, in the presence of chemical reaction and thermal radiation with variable surface conditions. Ali^29^ investigated free convection flow of an electrically conducting fluid along a vertical plate embedded in a thermally stratified porous medium in the presence of a uniform normal magnetic field. MHD mixed convection from a semi-infinite, isothermal, vertical and permeable surface immersed in a uniform porous medium in the presence of thermal radiation and Dufour and Soret effects are studied by Ali^30^. Sparrow et al.^31^ analysed the natural convective flow othrough inclined plates along with generation of longitudinal. vortices. Free convective flow passing through an inclined flat plate together with variable viscosity and internal heat generation are studied by Siddiqa et al.^32^. Bhuvaneswari et al.^33^ examined natural convection flow of an inclined plate with variable thermal conductivity by scaling group transformations. Some relevant investigations related to inclined plates regarding different fluid models are mentioned in these studies^34–38^.

In the previous investigation, Zafar et al.^39^ discussed the flow of Maxwell fluid over an inclined plate with heat and mass flux and computed solution by using the Laplace transformation method, because it has efficient applications for non-uniform boundary conditions and computed the exact solution for the presented model. But in the literature Oldroyd-B fluid model for inclined plate are not investigated yet nor published. To fill this gape a new Oldroyd-B model developed. For seeking exact solutions in terms of special functions namely Mittag–Leffler functions, G-function etc., for Oldroyd-B fluid velocity, concentration and Oldroyd-B fluid temperature, Laplace integral transformation method is used to solve the non-dimensional model. The contribution of different velocity components are considered as thermal, mass and mechanical, and analyse the impacts of these components on the fluid dynamics. For several physical significance of various fluidic parameters on Oldroyd-B fluid velocity, concentration and Oldroyd-B fluid temperature distributions are demonstrated through various graphs. Furthermore, for being validated the acquired solutions, some limiting models such as Newtonian fluid in the absence of different fluidic parameters. Moreover, the graphical representations of the analytical solutions illustrated the main results of the present work and studied various cases regarding the movement of plate.

## Problem statement

**Figure 1:**
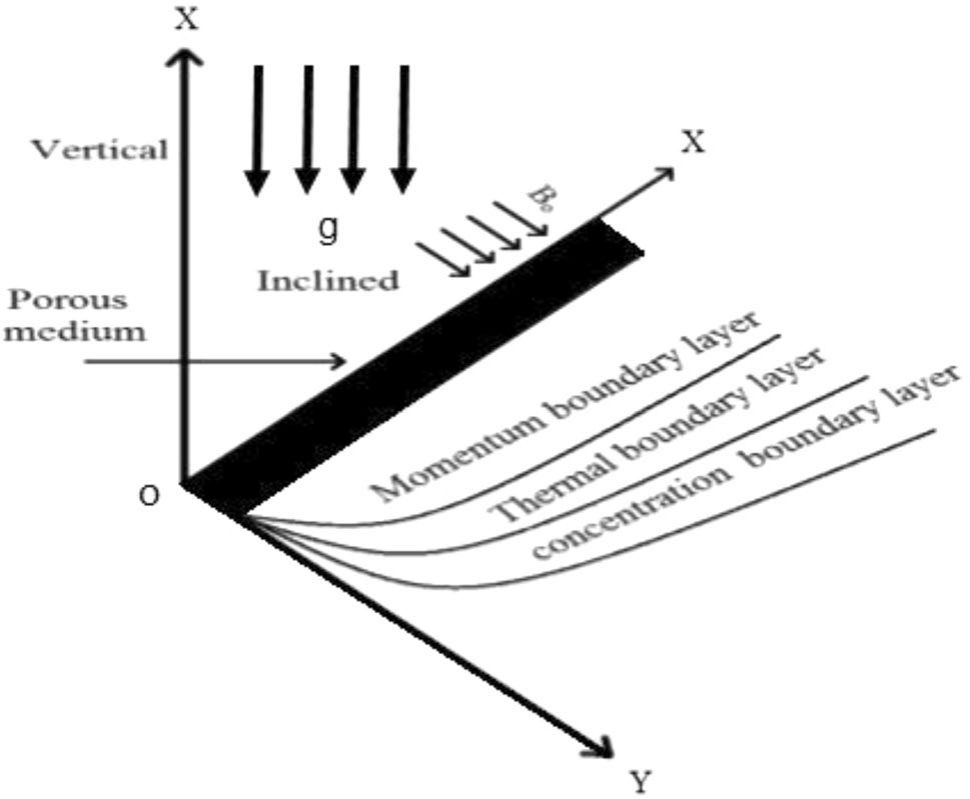
Geometry of the problem.

Consider the unsteady, incompressible MHD Oldroyd-B fluid flow through inclined plate, having length infinite, that is embedded in a porous media. The plate is considered at y = 0 and the fluid flow is restrained to y > 0, in the direction that is along to the plate. Also, assumed that x-axis is taken along vertical, and an angle $\upsilon$, $\left( {0 \le \upsilon \le \frac{\pi }{2}} \right)$ that the plate which make with the vertical, and supposed magnetic intensity $\overrightarrow {{B_{0} }} = (B_{0} \cos \wp ,B_{0} \sin \wp )$, where $\wp$ represents slanted angle between the magnetic lines and inclined plate having porous layered, together with the supposition that magnetic field is fixed regarding fluid or relative to the plate (as exhibited in Fig. 1). Initially, for time t = 0, the fluid and the plate both are in the static mode, having ambient temperature $T_{\infty }$ and concentration $C_{\infty }$. Later on, when time $t = 0^{ + }$, the plate begins to oscillate and fluid starts to move with with certain $Vg(t)$ in opposition, where $V$ represents characteristic velocity, and the wall temperature in theform $T_{\infty } + T_{w} f(t)$ and concentration $C_{w} .$ where $f( \cdot )$ and $g( \cdot )$ are continuous piecewise function. It is conceived that velocity, energy and concentration are functions of y and t only. The following principal equations for Oldroyd-B fluid under Boussinesq's approximation, for velocity field, concentration distribution and energy transfer are obtained as^33,34^:

1 $$\begin{aligned} \left( {1 + \lambda_{1} \frac{\partial }{\partial t}} \right)\frac{{\partial {\text{v}} (y,t)}}{\partial t} & = v\left( {1 + \lambda_{2} \frac{\partial }{\partial t}} \right)\frac{{\partial^{2} {\text{v(y,t)}}}}{{\partial y^{2} }} + \left( {1 + \lambda_{1} \frac{\partial }{\partial t}} \right)\left( {g\cos (\upsilon )\left( {T - T_{\infty } } \right)\beta_{T} + g\cos (\upsilon )\left( {C - C_{\infty } } \right)\beta_{C} } \right) \\ & \;\;\; - \left( {1 + \lambda_{1} \frac{\partial }{\partial t}} \right)\left( {\frac{\nu }{K}{\text{v}} (y,t) + \frac{{\sigma B_{0}^{2} \sin^{2} (\wp )}}{\rho }({\text{v}} \left( {y,t} \right) - \varepsilon Vg(t))} \right) \\ \end{aligned}$$

Also equations for heat and mass are taken as:

2 $$\begin{gathered} \rho c_{p} \frac{\partial T(y,t)}{{\partial t}} = k\frac{{\partial^{2} T(y,t)}}{{\partial y^{2} }} - \frac{{\partial q_{r} }}{\partial y}, \, \hfill \\ \left[ {q_{r} = - \frac{4}{3}\frac{{\sigma_{1} }}{{k_{R} }}\frac{{\partial T^{4} }}{\partial y},T^{4} \approx 4T_{\infty }^{3} T - 3T_{\infty }^{4} } \right], \hfill \\ \end{gathered}$$

3 $$\frac{\partial C(y,t)}{{\partial t}} = D_{C} \frac{{\partial^{2} C(y,t)}}{{\partial y^{2} }} - R_{C} \left( {C - C_{\infty } } \right),$$

with connected initial conditions together with boundary conditions are given below:

4 $${\text{v}} (0,t) = Vg(t),\;T(0,t) = T_{\infty } + T_{w} f(t);\,C(0,t) = C_{w} ;\,\,t > 0,$$

5 $${\text{v}} (y,t) < \infty ,\;T(y,t) \to T_{\infty } ;C(y,t) \to C_{\infty } \;{\text{as}}\;y \to \infty .$$

6 $${\text{v}} (y,0) = 0,\;T(y,0) = T_{\infty } ;\;C(y,0) = C_{\infty } ;\, \, y \ge 0,$$

where ${\text{v}} (y,t)$, $C(y,t)$ and $T(y,t)$ represents the fluid velocity, concentration andenegy distribution. Where the parameters $\beta_{T} ,$ $D_{C} ,$ $\beta_{C} ,$ $R_{C} ,$ $\lambda_{1} ,$ $c_{p} ,$ $g,$ $\sigma ,$ $\nu ,$ $q_{r} ,$ $\rho ,$ $k,$ and $\lambda_{2}$ are thermal expansion coefficient, mass diffusivity, concentration expansion coefficient, chemical reaction, relaxation time, specific heat at constant pressure, gravitational acceleration, electrical conductivity, kinematic viscosity, radiative heat flux, fluid density, thermal conductivity and retardation time. Also, the parameter $\varepsilon$ used in the velocity expression has values 0 and 1 for cases MFFRF and MFFRP respectively.

To reduce the number of involving parameters, introducing the following new set of unit-free quantities

7 $$\begin{aligned} t^{ * } & = \frac{{V^{2} }}{\nu }t,\,y^{ * } = \frac{V}{\nu }y,{\text{v}}^{ * } = \frac{\text{v}}{V},\,T^{ * } = \frac{{T - T_{\infty } }}{{T_{w} }},\,\lambda_{2}^{*} = \frac{{V^{2} }}{\nu }\lambda_{2} ,K^{*} = \left( {\frac{\nu }{V}} \right)^{2} \,\frac{1}{K},\,\,\lambda_{1}^{*} = \frac{{V^{2} }}{\nu }\lambda_{1} , \\ C^{ * } & = \frac{{C - C_{\infty } }}{{C_{w} - C_{\infty } }},\,\,f^{*} (t^{*} ) = f\left( {\frac{\nu }{{V^{2} }}t^{*} } \right),g^{*} (t^{*} ) = g\left( {\frac{\nu }{{V^{2} }}t^{*} } \right). \\ \end{aligned}$$

After employing this substitution, the transformed non-dimensional model, ignoring $*$ in the modified form, is written as:

8 $$\begin{aligned} \left( {1 + \lambda_{1} \frac{\partial }{\partial t}} \right)\frac{{\partial {\text{v(y,t)}}}}{\partial t} & = \left( {1 + \lambda_{2} \frac{\partial }{\partial t}} \right).\frac{{\partial^{2} {\text{v(y,t)}}}}{{\partial y^{2} }} + \left( {1 + \lambda_{1} \frac{\partial }{\partial t}} \right)\left( {T(y,t)\cos (\upsilon ) + NC(y,t)\cos (\upsilon )} \right) \\ & \;\;\; - \left( {1 + \lambda_{1} \frac{\partial }{\partial t}} \right)\left( {K{\text{v(y,t) + }}M\sin^{2} (\wp )\left( {{\text{v(y,t)}} - \varepsilon f(t)} \right)} \right),\,\, \\ \end{aligned}$$

9 $$\frac{{\partial^{2} T(y,t)}}{{\partial y^{2} }} = \Pr_{eff} \left( {\frac{\partial T(y,t)}{{\partial t}}} \right),$$

10 $$\frac{{\partial^{2} C(y,t)}}{{\partial y^{2} }} = Sc.\left( {\frac{\partial C(y,t)}{{\partial t}} + R_{C} C(y,t)} \right),$$

for $t,y > 0$, the conditions for modified model is

11 $${\text{v}} (0,t) = g(t);T(0,t) = f(t);\,C(0,t) = 1;\,\,\,t > 0,$$

12 $${\text{v}} (y,t) < \infty ,\,\,T(y,t) \to 0,C(y,t) \to 0\;{\text{as}}\;y \to \infty ,$$

13 $${\text{v}} (y,0) = C(y,0) = T(y,0) = 0{\text{ for }}y \ge 0,$$

where $$\Pr_{eff} = \frac{\Pr }{{1 + N_{r} }}$$,$$\,\,N_{r} = \frac{16}{3}\frac{{{\upsigma }_{1} }}{{kk_{R} }}T_{\infty }^{3}$$, $$\Pr = \frac{{\mu c_{p} }}{k}$$,$$N = \frac{{\beta_{C} (C_{w} - C_{\infty } )}}{{\beta_{T} T_{w} }},\,\,\,M = \frac{{\sigma B_{0}^{2} }}{\rho }\frac{\nu }{{V^{2} }},\,\,Sc = \frac{\nu }{{D_{C} }}.$$

Here, $\Pr_{eff}$ represents effective Prandtl number, $\,\,N_{r}$ denotes radiation conduction parameter, $\Pr$ is denoted by Prandtl number, $N$(the ratio of buoyancy forces) while $M$ denotes the magnetic parameter, $Sc$ represents schmidt number and and $R_{C}$ denoted chemical reaction parameter. $k_{1}$ is the coefficient of Rosseland absorption, $\sigma_{1}$ Stefan-Boltzmann constant, $q_{r}$ is known as radiative heat flux and $K$ is defined as porosity.

## Solution of the problem

In this section solution of the proposed model will be computed. To find the velocity field solution, first we acquired the temperature and concentration solution. In contemplation of, finding the exact expresssions for the temperature and concentration from Eqs. (9), (10), we apply the standard Laplace transform method.

### Solution of temperature field

Employing Laplace transformation to Eq. (9) with the stated set of conditions, we obtained

14 $$\frac{{d^{2} \overline{T} (y,q)}}{{dy^{2} }} - q\Pr_{eff} \overline{T} (y,q) = 0.$$

With the stated conditions for temperature as

15 $$\overline{T} (0,q) = \overline{f} (q){\text{ and }}\overline{T} (y,q) \to 0{\text{ as y}} \to \infty {.}$$

The solution is written as

16 $$\overline{T} (y,q) = c_{1} e^{{y\sqrt {\Pr_{eff} q} }} + c_{2} e^{{ - y\sqrt {\Pr_{eff} q} }}$$

Using the conditions as given in Eq. (15), then the solution of Eq. (16), is written as

17 $$\overline{T} (y,q) = \left\{ \begin{gathered} \overline{f} (q){ ; }y = 0 \hfill \\ \overline{f} (q)e^{{ - y\sqrt {\Pr_{eff} q} }} {; }y > 0 \hfill \\ \end{gathered} \right.$$

Employing the Laplace inverse transformation on Eq. (17)

18 $$T(y,t) = \left\{ \begin{gathered} f(t){ ; }y = 0 \hfill \\ \frac{y}{2}\sqrt {\frac{{\Pr_{eff} }}{\pi }} \int\limits_{0}^{t} {f(t - \eta )} \frac{{e^{{ - \left( {\frac{{\Pr_{eff} y^{2} }}{4\eta }} \right)}} }}{{\eta^{{\tfrac{3}{2}}} }}d\eta ; \, y > 0 \hfill \\ \end{gathered} \right.$$

with applying the following relation

19 $$L^{ - 1} \left\{ {e^{ - f\sqrt q } } \right\} = \frac{f}{{2\sqrt {\pi t^{3} } }}.e^{{ - \tfrac{1}{4t}f^{2} }} { ; (}f > 0{)}.\,$$

Further, the rate of heat transfer known as Nusselt number, it is denoted by (Nu) and can be calculated mathematically as:

20 $$\begin{aligned} Nu & = - \left. {\frac{\partial T(y,t)}{{\partial y}}} \right|_{y = 0} \\ Nu & = \frac{1}{2}\sqrt {\frac{{\Pr_{eff} }}{\pi }} \int\limits_{0}^{t} {\frac{f(t - \eta )}{{\eta^{{\tfrac{3}{2}}} }}} d\eta ,\, \\ \end{aligned}$$

### Solution of concentration field

Using Laplace transformation technique on Eq. (10) with corresponding stated conditions,we have

21 $$\frac{{d^{2} \overline{C} (y,q)}}{{dy^{2} }} - Sc(q + R_{C} )\overline{C} (y,q) = 0.$$

with the stated conditions for concentration written as

22 $$\overline{C} (y,q) \to 0{\text{ as y}} \to \infty ,{\text{ and }}\overline{C} (0,q) = \frac{1}{q}$$

The solution is written as

23 $$\overline{C} (y,q) = c_{3} e^{{ - y\sqrt {Sc(q + R_{C} )} }} + c_{4} e^{{y\sqrt {Sc(q + R_{C} )} }}$$

Applying the conditions as given in Eq. (22), its solution is written

24 $$\overline{C} (y,q) = \frac{1}{q}e^{{ - y\sqrt {Sc(q + R_{C} )} }}$$

To find $C(y,t)$, employing Laplace inverse transformation on Eq. (24), to acquire the required solution in the form

25 $$C(y,t) = \frac{1}{2}\left[ {e^{{ - y\sqrt {ScR_{C} } }} erfc\left( {\frac{y}{2}\sqrt{\frac{Sc}{t}} - \sqrt {R_{C} t} } \right) + e^{{y\sqrt {ScR_{C} } }} erfc\left( {\frac{y}{2}\sqrt{\frac{Sc}{t}} + \sqrt {R_{C} t} } \right)} \right].$$

with applying the following relation

26 $$L^{ - 1} \left\{ {\frac{{e^{{ - f\sqrt {q + \partial } }} }}{q - b}} \right\} = \frac{{{\text{e}}^{bt} }}{2}\left[ {{\text{e}}^{{ - f\sqrt {\partial + b} }} erfc\left( {\frac{f}{2\sqrt t } - \sqrt {(\partial + b)t} } \right) + {\text{e}}^{{f\sqrt {\partial + b} }} erfc\left( {\frac{f}{2\sqrt t } + \sqrt {(\partial + b)t} } \right)} \right].\,$$

Moreover, the rate of masstransfer is known as Sherwood number, it is denoted by (Sh) and mathematically it is defined as:

27 $$Sh = - \left. {\frac{\partial C(y,t)}{{\partial y}}} \right|_{y = 0}$$

Solving Eqs. (25) and (27), we obtained

28 $$Sh = \sqrt {Sc} \left[ {\frac{{e^{{ - tR_{C} }} }}{\sqrt \pi } + \frac{1}{{\sqrt {R_{C} } }}erf\left( {\sqrt {R_{C} t} } \right)} \right].$$

### Solution of velocity field

To derive the solution for velocity field Eq. (8) with appropriate non-dimensional conditions Eqs. (11)–(13), employing the Laplace transformation method, we obtained

29 $$\begin{aligned} \left( {q + \lambda_{1} q^{2} } \right)\overline{v}(y,q) & = \left( {1 + \lambda_{2} q} \right)\frac{{d^{2} \overline{v}(y,q)}}{{dy^{2} }} + \left( {1 + \lambda_{1} q} \right)\left( {N\overline{C}(y,q)\cos (\upsilon ) + \overline{T}(y,q)\cos (\upsilon )} \right) \\ & \;\;\; - \left( {1 + \lambda_{1} q} \right)\left( {K\overline{v}(y,q) + M\sin^{2} (\wp )\left( {\overline{v}(y,q) - \varepsilon \overline{g} (q)} \right)} \right), \\ \end{aligned}$$

with conditions defined for momentum equation

30 $$\overline{v}(0,q) = \overline{g} (q),\;\overline{v}(y,q) < \infty \;{\text{as}}\;y \to \infty .$$

where $\overline{g} (q)$ and $\overline{v}(y,q)$ represents Laplace transformation of $g(t)$ and ${\text{v}}(y,t)$ respectively. Substituting, the expressions for temperature from Eq. (19) and the concentration from (25) into (29), it gives

31 $$\begin{gathered} \left( {1 + \lambda_{2} q} \right)\frac{{d^{2} \overline{v}(y,q)}}{{dy^{2} }} - \left( {\lambda_{1} q^{2} + \left( {1 + \lambda_{1} \Lambda } \right)q + \Lambda } \right)\overline{v}(y,q) \hfill \\ \;\;\; = - \left( {1 + \lambda_{1} q} \right)\left( {\varepsilon M\,\sin^{2} (\wp )\overline{g} (q) + \cos (\upsilon )\overline{f} (q)e^{{ - y\sqrt {\Pr_{eff} \,q} }} + N\frac{1}{q}{\text{e}}^{{ - y\sqrt {Sc.(R_{C} + q)} }} \cos (\upsilon )} \right). \hfill \\ \end{gathered}$$

where $$\Lambda = M\,\sin^{2} (\wp ) + K$$.

The obtained solution of (31) after using the boundry conditions mentioned in Eq. (30), is

32 $$\begin{aligned} \overline{v}(y,q) & = \, \overline{{\text{g}}} (q){\text{e}}^{{ - y\sqrt {\frac{{\lambda_{1} q^{2} + \left( {1 + \lambda_{1} \Lambda } \right)q + \Lambda }}{{1 + \lambda_{2} q}}} }} + \frac{{\varepsilon M\sin^{2} (\wp ).\left( {1 + \lambda_{1} q} \right).\overline{{\text{g}}} (q)}}{{\lambda_{1} q^{2} + \left( {1 + \lambda_{1} \Lambda } \right).q + \Lambda }}.\left( {1 - {\text{e}}^{{ - y\sqrt {\frac{{\lambda_{1} q^{2} + \left( {1 + \lambda_{1} \Lambda } \right).q + \Lambda }}{{1 + \lambda_{2} q}}} }} } \right) \\ & \,\,\, - \frac{{\left( {1 + \lambda_{1} q} \right)\overline{{\text{f}}} (q)\cos (\upsilon )}}{{\left( {q + \lambda_{2} q^{2} } \right)\Pr_{eff} - \left( {\lambda_{1} q^{2} + \left( {1 + \lambda_{1} \Lambda } \right).q + \Lambda } \right)}}\left( {{\text{e}}^{{ - y\sqrt {\Pr_{eff} } \sqrt q }} - {\text{e}}^{{ - y\sqrt {\frac{{\lambda_{1} q^{2} + \left( {1 + \lambda_{1} \Lambda } \right).q + \Lambda }}{{1 + \lambda_{2} q}}} }} } \right) \\ & \;\;\, - \frac{1}{q}.\frac{N\cos (\upsilon )}{{\left( {(q + R_{C} )\left( {1 + \lambda_{2} q} \right)Sc - \left( {\lambda_{1} q^{2} + \left( {1 + \lambda_{1} \Lambda } \right).q + \Lambda } \right)} \right)}}\left( {{\text{e}}^{{ - y\sqrt {Sc} \sqrt {(q + R_{C} )} }} - {\text{e}}^{{ - y\sqrt {\frac{{\lambda_{1} q^{2} + \left( {1 + \lambda_{1} \Lambda } \right).q + \Lambda }}{{1 + \lambda_{2} q}}} }} } \right). \\ \end{aligned}$$

The velocity field solution can be written as in the following form

33 $${\text{v}}(y,t) = {\text{v}}_{T} (y,t) + {\text{v}}_{m} (y,t) + {\text{v}}_{C} (y,t),$$

where

34 $$\begin{aligned} {\text{v}}_{m} (y,t) & = \int\limits_{0}^{t} {\omega_{1} (y,s;\Lambda ,\lambda_{1} ,\lambda_{2} ).g\left( {t - s} \right)ds} \\ & \;\; + \varepsilon M\sin^{2} (\wp )\int\limits_{0}^{t} {\omega_{2} (s;\Lambda ,\lambda_{1} ).g(t - s)ds} \\ & \;\; - \varepsilon M\sin^{2} (\wp ).\int\limits_{0}^{t} {\int\limits_{0}^{s} {\omega_{2} (u;\Lambda ,\lambda_{1} )} } \omega_{1} (y,s - u;\Lambda ,\lambda_{1} ,\lambda_{2} )g\left( {t - s} \right)duds, \\ \end{aligned}$$

35 $$\begin{aligned} {\text{v}}_{T} (y,t) & = - \cos (\upsilon )\left[ {\int\limits_{0}^{t} {\int\limits_{0}^{s} {\omega_{3} (t - s;\Lambda ,\lambda_{1} ,\lambda_{2} ,\Pr_{eff} )} \left( {\frac{y}{{2\sqrt {\pi u^{3} } }}e^{{ - \frac{{y^{2} }}{4u}}} } \right)f(s - u)du} } \right.ds \\ & \;\; - \int\limits_{0}^{t} {\int\limits_{0}^{s} {\omega_{3} (t - s;\Lambda ,\lambda_{1} ,\lambda_{2} ,\Pr_{eff} )\omega_{1} (y,u;\Lambda \left. {,\lambda_{1} ,\lambda_{2} )f(s - u)duds} \right]} } , \\ \end{aligned}$$

36 $$\begin{gathered} {\text{v}}_{C} (y,t) = N\cos (\upsilon )(\int\limits_{0}^{t} {\omega_{5} (y,s;\lambda_{1} ,\lambda_{2} ,\Lambda )\omega_{4} (t - s;\lambda_{1} ,\lambda_{2} ,S_{c} ,R_{C} ,\Lambda )ds} \hfill \\ \, - \int\limits_{0}^{t} {\phi \left( {y\sqrt {Sc} ,s;R_{C} ,0} \right)} \omega_{4} (t - s;\lambda_{1} ,\lambda_{2} ,S_{c} ,R_{C} ,\Lambda )ds). \hfill \\ \end{gathered}$$

Here ${\text{v}}_{m} (y,t)$ dentes mechanical, where ${\text{v}}_{C} (y,t){\text{ and v}}_{T} (y,t)$ represents concentration and thermal components of velocity respectively.

Also, the inverse of some terms used in the three components are calculated in the following way:

$$\begin{aligned} \omega_{1} \left( {y,t;\Lambda ,\lambda_{1} ,\lambda_{2} } \right) & = L^{ - 1} \left\{ {{\text{e}}^{{ - y\sqrt {\frac{{\Lambda + \lambda_{1} q^{2} + \left( {1 + \lambda_{1} \Lambda } \right)q}}{{1 + \lambda_{2} q}}} }} } \right\} \\ & = \sum\limits_{k = 0}^{\infty } {\sum\limits_{l = 0}^{\infty } {\sum\limits_{m = 0}^{\infty } {\tfrac{{( - y)^{2k + 2l + 2m} .(\Lambda )^{k + m} .(\lambda_{1} )^{m} .(1 + \lambda_{1} \Lambda )^{l} .\Gamma (k + l + m + 1)}}{{l!m!.(\lambda_{2} )^{k + l + m} .\Gamma (2k + 2l + 1).\Gamma (k + m + 1)}}} } } .(t)^{k - 1} E_{1,k}^{k + l + m} ( - \tfrac{1}{{\lambda_{2} }} \cdot t) \\ \end{aligned}$$,$$\begin{aligned} \omega_{2} (t;\lambda_{1} ,\Lambda ) & = L^{ - 1} \left\{ {\frac{{1 + \lambda_{1} q}}{{\lambda_{1} q^{2} + \left( {1 + \lambda_{1} \Lambda } \right)q + \Lambda }}} \right\} \\ & = \sum\limits_{\ell = 0}^{\infty } {\tfrac{{( - \Lambda )^{\ell } }}{{(\lambda_{1} )^{\ell + 1} }}} .\left( {G_{1, - \ell - 1,\ell + 1} ( - (\Lambda + \tfrac{1}{\lambda 1}),t) + \lambda_{1} G_{1, - \ell ,\ell + 1} ( - (\Lambda + \tfrac{1}{\lambda 1}),t)} \right), \\ \end{aligned}$$

$$\begin{aligned} \omega_{3} (t;\lambda_{1} ,\lambda_{2} ,\Pr_{eff} ,\Lambda ) & = L^{ - 1} \left\{ {\frac{{1 + \lambda_{1} q}}{{(\lambda_{2} \Pr_{eff} - \lambda_{1} )q^{2} + \left( {\Pr_{eff} - 1 - \lambda_{1} \Lambda } \right)q - \Lambda }}} \right\} \\ & = \sum\limits_{\beta = 0}^{\infty } {\tfrac{{(\Lambda )^{\beta } }}{{(\lambda_{2} \Pr_{eff} - \lambda_{1} )^{\beta + 1} }}} .\left( {G_{1, - \beta - 1,\beta + 1} ( - \tfrac{{\Pr_{eff} - 1 - \lambda_{1} \Lambda }}{{\lambda_{2} \Pr_{eff} - \lambda_{1} }},t) + \lambda_{1} G_{1, - \beta ,\beta + 1} ( - \tfrac{{\Pr_{eff} - 1 - \lambda_{1} \Lambda }}{{\lambda_{2} \Pr_{eff} - \lambda_{1} }},t)} \right), \\ \end{aligned}$$

$$\begin{aligned} \omega_{4} (t;\lambda_{1} ,\lambda_{2} ,S_{c} ,R_{C} ,\Lambda ) & = L^{ - 1} \left\{ {\frac{{1 + \lambda_{1} q}}{{(\lambda_{2} S_{c} - \lambda_{1} )q^{2} + \left( {S_{c} (1 + \lambda_{2} R_{C} ) - (1 + \lambda_{1} \Lambda )} \right)q + (S_{c} R_{C} - \Lambda )}}} \right\} \\ & = \sum\limits_{\eta = 0}^{\infty } {\tfrac{{(\Lambda - S_{c} R_{C} )^{\eta } }}{{(\lambda_{2} S_{c} - \lambda_{1} )^{\eta + 1} }}} .\left( {G_{1, - \eta - 1,\eta + 1} ( - \tfrac{{S_{c} (1 + \lambda_{2} R_{C} ) - (1 + \lambda_{1} \Lambda )}}{{\lambda_{2} S_{c} - \lambda_{1} }},t) + \lambda_{1} G_{1, - \eta ,\eta + 1} ( - \tfrac{{S_{c} (1 + \lambda_{2} R_{C} ) - (1 + \lambda_{1} \Lambda )}}{{\lambda_{2} S_{c} - \lambda_{1} }},t)} \right), \\ \end{aligned}$$

37 $$\begin{aligned} \omega_{5} (y,t;\Lambda ,\lambda_{1} ,\lambda_{2} ) & = L^{ - 1} \left\{ {\frac{{{\text{e}}^{{ - y\sqrt {\frac{{\Lambda + \lambda_{1} q^{2} + \left( {1 + \lambda_{1} \Lambda } \right)q}}{{1 + \lambda_{2} q}}} }} }}{q}} \right\} \\ & = \sum\limits_{k = 0}^{\infty } {\sum\limits_{l = 0}^{\infty } {\sum\limits_{m = 0}^{\infty } {\tfrac{{( - y)^{2k + 2l + 2m} .(\Lambda )^{k + m} .(\lambda_{1} )^{m} .(1 + \lambda_{1} \Lambda )^{l} .\Gamma (k + l + m + 1)}}{{l!m!.(\lambda_{2} )^{k + l + m} .\Gamma (2k + 2l + 1).\Gamma (k + m + 1)}}} } } .(t)^{k} E_{1,k - 1}^{k + l + m} ( - \tfrac{1}{{\lambda_{2} }} \cdot t). \\ \end{aligned}$$

where the Laplace inverse of expressions in the form of generalized G-function and Mittag–Leffler function are defined as:

$$\begin{aligned} L^{ - 1} \left\{ {\frac{{\rho^{x} }}{{(\rho^{z} - h)^{d} }}} \right\} & = G_{z,x,d} (h,t);{\text{Re}} (zd - x),{\text{Re}} (\rho ) > 0,|\tfrac{h}{{\rho^{z} }}| < 1, \\ G_{z,x,d} (h,t) & = \sum\limits_{\ell = 0}^{\infty } {\frac{{h^{\ell } \Gamma (d + \ell )}}{\Gamma (d)\Gamma (\ell + 1)}.\frac{{t^{(d + \ell )z - x - 1} }}{\Gamma ((d + \ell )z - x)}} \\ \end{aligned}$$

and $$L^{ - 1} \left\{ {\frac{{\rho^{zd - x} }}{{(\rho^{z} - h)^{d} }}} \right\} = t^{x - 1} E_{z,x}^{d} (ht^{z} ).$$

Also, assigning $g( \cdot )$ and $f( \cdot )$ in different appropriate forms, analytical solution for various types of moving fluid, that is discussed in 4th section, are recovered with technical relevance. From the velocity solution ${\text{v}} (y,t)$ computed in Eq. (33), which can easily satisfies the imposed initial / boundary conditions.

When y tends to infinit, as $y \to \infty$, the velocity becomes:

38 $$\mathop {\lim }\limits_{y \to \infty } \,{\text{v}} (y,t) = \left\{ \begin{gathered} 0\,\,\,\,\,\,\,\,\,\,\,\,\,\,\,\,\,\,\,\,\,\,\,\,\,\,\,\,\,\,\,\,\,\,\,\,\,\, \, \, \, \,if\,\,\varepsilon = 0 \hfill \\ M\sin^{2} (\wp )\int\limits_{0}^{t} {\omega_{2} (s;\Lambda ,\lambda_{1} ) \cdot g(t - s)ds} \, \,if\,\,\varepsilon = 1. \hfill \\ \end{gathered} \right.$$

Finally, for the case magnetic flux is fixed relative to plate (MFFRP), it is observed that the fluid does not remain at static position when it far away from the plate.

It is perceived that the movement of the fluid are effected through the transfer of heat and mass in various engineering applications. So, different cases regarding fluid motion when the influence of some fluidic parameters are ignored, discussed in the next section for physical properties of computed results.

## Different cases relating the motion of the plate

In the present section, different cases regarding the modes of heating plate are discussed and the corresponding solutions are calculated for each case.

### Case-I: $f(t) = H(t)$ (for constant heating plate)

Replacing $f(t) = H(t)$ in Eq. (35), we obtained

39 $$\begin{aligned} {\text{v}}_{{T_{con} }} (y,t) & = - \cos (\upsilon )\left[ {\int\limits_{0}^{t} {\left( {\frac{y}{{2\sqrt {\pi s^{3} } }}e^{{ - \frac{{y^{2} }}{4s}}} } \right)\omega_{3} \left( {t - s;\Lambda ,\lambda_{1} ,\lambda_{2} ,\Pr_{eff} } \right)} ds} \right. \\ & \;\;\; \, \left. { - \int\limits_{0}^{t} {\omega_{1} (y,s;\Lambda ,\lambda_{1} ,\lambda_{2} )\omega_{3} (t - s;\Lambda ,\lambda_{1} ,\lambda_{2} ,\Pr_{eff} )ds} } \right], \\ \end{aligned}$$

### Case-II: $f(t) = H(t)\left( {1 - ae^{ - bt} } \right)$ (for exponential heating plate)

Now, replacing $f(t) = H(t)\left( {1 - ae^{ - bt} } \right)$ where $0 < b < a < 1$, in Eq. (35), we have

40 $$\begin{aligned} {\text{v}}_{{T_{\exp } }} (y,t) & = - \cos (\upsilon )\int\limits_{0}^{t} {\omega_{3} (s;\Lambda ,\lambda_{1} ,\lambda_{2} ,\Pr_{eff} )} erfc\left( {\frac{y}{{2\sqrt {t - s} }}} \right)ds \\ & \;\; + a\cos (\upsilon )\int\limits_{0}^{t} {\omega_{3} (s;\Lambda ,\lambda_{1} ,\lambda_{2} ,\Pr_{eff} )} \phi \left( {y\sqrt {\Pr_{eff} } ,t - s;0, - b} \right)ds \\ & \;\; + \cos (\upsilon )\int\limits_{0}^{t} {\int\limits_{0}^{s} {\omega_{1} (y,u;\Lambda ,\lambda_{1} ,\lambda_{2} )\omega_{3} (t - s;\Lambda ,\lambda_{1} ,\lambda_{2} ,\Pr_{eff} )duds} } \\ & \;\; - a\cos (\upsilon )\int\limits_{0}^{t} {\int\limits_{0}^{s} {\omega_{3} (t - s;\Lambda ,\lambda_{1} ,\lambda_{2} ,\Pr_{eff} )e^{ - bu} \omega_{1} (y,s - u;\Lambda ,\lambda_{1} ,\lambda_{2} )duds} } , \\ \end{aligned}$$

### Case-III: $g(t) = H(t)t^{\alpha }$ (for accelerating the plate)

Now, substituting $g(t) = H(t)t^{\alpha } ,$ with $\alpha > 0$, into Eq. (34), we obtained

41 $$\begin{aligned} {\text{v}}_{\alpha m} (y,t) & = \int\limits_{0}^{t} {\omega_{1} (y,s;\Lambda ,\lambda_{1} ,\lambda_{2} ).(t - s)^{\alpha } ds + } \\ & \;\; + \varepsilon M\sin^{2} (\wp )\int\limits_{0}^{t} {\omega_{2} (s;\Lambda ,\lambda_{1} ) \cdot (t - s)^{\alpha } ds} \\ & \;\; - \varepsilon M \cdot \int\limits_{0}^{t} {\int\limits_{0}^{s} {(t - s)^{\alpha } \omega_{2} (u;\Lambda ,\lambda_{1} )} } \cdot \omega_{1} (y,s - u;\Lambda ,\lambda_{1} ,\lambda_{2} )duds, \\ \end{aligned}$$

that represented the fluid motions due to a constantly, slowly or highly accelerating the plate.

Additionally, the case when $\alpha = 0,$ i.e., movement in the plate for constant velocity. when $g(t) = H(t)$ then

42 $$\begin{aligned} {\text{v}}_{0m} (y,t) & = \int\limits_{0}^{t} {\omega_{1} (y,s;\lambda_{1} ,\lambda_{2} ,\Lambda )ds} \\ & \;\;\; - \varepsilon M\sin^{2} (\wp )\int\limits_{0}^{t} {\int\limits_{0}^{s} {\omega_{2} (u;\lambda_{1} ,\Lambda )} } \omega_{1} (y,s - u;\lambda_{1} ,\lambda_{2} ,\Lambda )duds, \\ \end{aligned}$$

Further, by taking limit $\lambda_{1} ,\lambda_{2} \to 0,\upsilon = 0,K = 0{\text{ and }}\wp = \tfrac{\pi }{2}$ for Eq. (40), yields

43 $${\text{v}}_{m} (y,t) = \frac{y}{2\sqrt \pi }\int\limits_{0}^{t} {\frac{{(t - s)^{\alpha } }}{s\sqrt s }\exp \left( { - \frac{{y^{2} }}{4s} - \Lambda s} \right)ds + \varepsilon M\sin^{2} (\wp )\int\limits_{0}^{t} {(t - s)^{\alpha } e^{ - \Lambda s} erf\left( {\frac{y}{2\sqrt s }} \right)ds} } ,$$

the solutions for moving vertical plate in case of viscous fluid^25^.

### Case-IV: $g(t) = \cos (\omega t)\,H(t)\,{\text{or}}\,\,\sin (\omega t)H(t)$ (for oscillating the plate)

Substituting, $g(t) = \cos (\omega t)\,H(t)\,{\text{or}}\,\,\sin (\omega t)H(t)$, into Eq. (34), then solution becomes:

44 $$\begin{aligned} {\text{v}}_{cm} (y,t) & = \, \int\limits_{0}^{t} {\omega_{1} (y,s;\lambda_{1} ,\lambda_{2} ,\Lambda )\cos \left( {\omega \left( {t - s} \right)} \right)ds + \varepsilon M\sin^{2} (\wp )\left( {\frac{{\Lambda e^{ - \Lambda t} }}{{\Lambda^{2} + \omega^{2} }} + \frac{{\cos \left( {\omega t + \theta } \right)}}{{\sqrt {\Lambda^{2} + \omega^{2} } }}} \right)} \\ & \;\; - \varepsilon M\sin^{2} (\wp )\int\limits_{0}^{t} {\left( {\frac{{\Lambda e^{ - \Lambda s} }}{{\Lambda^{2} + \omega^{2} }} + \frac{{\cos \left( {\omega s + \theta } \right)}}{{\sqrt {\Lambda^{2} + \omega^{2} } }}} \right)} \omega_{1} (y,t - s;\lambda_{1} ,\lambda_{2} ,\Lambda )ds, \\ \end{aligned}$$

45 $$\begin{gathered} {\text{v}}_{sm} (y,t) = \, \int\limits_{0}^{t} {\omega_{1} (y,s;\lambda_{1} ,\lambda_{2} ,\Lambda )\sin \left[ {\omega (t - s)} \right]ds + \varepsilon M\sin^{2} (\wp )\left( {\frac{{\omega e^{ - \Lambda t} }}{{\Lambda^{2} + \omega^{2} }} + \frac{{\sin \left( {\omega t - \theta } \right)}}{{\sqrt {\Lambda^{2} + \omega^{2} } }}} \right)} \hfill \\ \,\,\,\,\,\,\,\,\,\, \, \, - \varepsilon M\sin^{2} (\wp )\int\limits_{0}^{t} {\left( {\frac{{\omega e^{ - \Lambda s} }}{{\Lambda^{2} + \omega^{2} }} + \frac{{\sin \left( {\omega s - \theta } \right)}}{{\sqrt {\Lambda^{2} + \omega^{2} } }}} \right)} \omega_{1} (y,t - s;\lambda_{1} ,\lambda_{2} ,\Lambda )ds. \hfill \\ \end{gathered}$$

Again limit $\lambda_{1} ,\lambda_{2} \to 0,\upsilon = 0,K = 0,\upsilon = 0{\text{ and }}\wp = \tfrac{\pi }{2}$, gives the suitable relevant results for Newtonian fluid^25^, written as

46 $${\text{v}}_{cm} (y,t) = \frac{y}{2\sqrt \pi }\int\limits_{0}^{t} {\frac{{\cos \left[ {\omega (t - s)} \right]}}{s\sqrt s }\exp \left( { - \frac{{y^{2} }}{4s} - \Lambda s} \right)ds + \varepsilon M\int\limits_{0}^{t} {\cos \left[ {\omega (t - s)} \right]e^{ - \Lambda s} erf\left( {\frac{y}{2\sqrt s }} \right)ds} } ,$$

47 $${\text{v}}_{sm} (y,t) = \frac{y}{2\sqrt \pi }\int\limits_{0}^{t} {\frac{{\sin \left[ {\omega (t - s)} \right]}}{s\sqrt s }\exp \left( { - \frac{{y^{2} }}{4s} - \Lambda s} \right)ds + \varepsilon M\int\limits_{0}^{t} {\sin \left[ {\omega (t - s)} \right]e^{ - \Lambda s} erf\left( {\frac{y}{2\sqrt s }} \right)ds.} }$$

It is depicted that, Eqs. (44) and  (45) that represens the non-dimensional form of the velocities ${\text{v}}_{cm} (y,t)$ and ${\text{v}}_{sm} (y,t)$ which represents the fluid motion after motion start initially. But, some time later, when the transients departs, then Eqs. (44) and  (45) becomes

48 $$\begin{aligned} {\text{v}}_{cmp} (y,t) & = \int\limits_{0}^{\infty } {\omega_{1} (y,s;\Lambda ,\lambda_{1} ,\lambda_{2} ).\cos \left( {\omega \left( {t - s} \right)} \right)ds + \frac{{\varepsilon M\sin^{2} (\wp )}}{{\sqrt {\omega^{2} + \Lambda^{2} } }}.\cos \left( {\omega t + \theta } \right)} \\ & \;\;\,\, - \frac{{\varepsilon M\sin^{2} (\wp )}}{{\sqrt {\omega^{2} + \Lambda^{2} } }}\int\limits_{0}^{\infty } {\cos \left( {\omega s + \theta } \right)} \,\omega_{1} (y,t - s;\Lambda ,\lambda_{1} ,\lambda_{2} )ds, \\ \end{aligned}$$

49 $$\begin{aligned} {\text{v}}_{smp} (y,t) & = \int\limits_{0}^{\infty } {\omega_{1} (y,s;\Lambda ,\lambda_{1} ,\lambda_{2} ).\sin \left[ {\omega (t - s)} \right]ds + \frac{{\varepsilon M\sin^{2} (\wp )}}{{\sqrt {\omega^{2} + \Lambda^{2} } }}.\sin \left( {\omega t - \theta } \right)} \\ & \;\,\,\, - \frac{{\varepsilon M\sin^{2} (\wp )}}{{\sqrt {\omega^{2} + \Lambda^{2} } }}\int\limits_{0}^{\infty } {\sin \left( {\omega s - \theta } \right)\,} \omega_{1} (y,t - s;\Lambda ,\lambda_{1} ,\lambda_{2} )ds, \\ \end{aligned}$$

The steady-state solution expressions.

Moreover, the governing Eq. (13) and the boundary conditions that can be verified easily from these solutions when the concentration distribution and thermal effects have been ignored. As a result, without regarding these effects, Eqs. (48) and (49) represents a certain specific time fluids flow. Now, Eqs. (48) and (49) after applying the limit as $y \to \infty$, are obtained and written as:

50 $${\text{v}}_{cmp} (\infty ,t) = \left\{ \begin{gathered} 0\,\,\,\,\,\,\,\,\,\,\,\,\,\,\,\,\,\,\,\,\,\,\,\,\,\,\,\,\,\,\,\,\,\,\,\,\,\,\,\,\,\,\,\,\,\,\,\,\,if\,\,\varepsilon = 0 \hfill \\ \frac{{M\sin^{2} (\wp )}}{{\sqrt {\Lambda^{2} + \omega^{2} } }}\cos (\omega t - \theta )\,\,\,\,if\,\,\,\varepsilon = 1, \hfill \\ \end{gathered} \right.$$

respectively

51 $${\text{v}}_{smp} (\infty ,t) = \left\{ \begin{gathered} 0\,\,\,\,\,\,\,\,\,\,\,\,\,\,\,\,\,\,\,\,\,\,\,\,\,\,\,\,\,\,\,\,\,\,\,\,\,\,\,\,\,\,\,\,\,\,\,\,\,if\,\,\varepsilon = 0 \hfill \\ \frac{{M\sin^{2} (\wp )}}{{\sqrt {\Lambda^{2} + \omega^{2} } }}\sin (\omega t - \varphi )\,\,\,\,\,if\,\,\,\varepsilon = 1. \hfill \\ \end{gathered} \right.$$

Next, if we substitute $g(t) = H(t)\sin (\omega t)\,\,{\text{or}}\,\,g(t) = {\text{H(t)cos}}(\omega t)$ in Eq. (38). Then related results

52 $${\text{v}}_{c} (\infty ,t) = \left\{ \begin{gathered} \, 0\,\,\,\,\,\,\,\,\,\,\,\,\,\,\,\,\,\,\,\,\,\,\,\,\,\,\,\,\,\,\,\,\,\,\,\,\,\,\,\,\,\,\,\,\,\,\,\,\,\,\,\,\,\,\,\,\,\,\,\,\,\,\,\,\,\,\,\,\,\,\,\,\,\,\,\,\,\,\,\,\,\,\, \, \,\,if\,\,\varepsilon = 0 \hfill \\ - \frac{{M^{2} \sin^{4} (\wp )}}{{\Lambda^{2} + \omega^{2} }}e^{ - \Lambda t} + \frac{{M\sin^{2} (\wp )}}{{\sqrt {\Lambda^{2} + \omega^{2} } }}\cos (\omega t - \theta )\,\,\,\,if\,\,\,\varepsilon = 1, \hfill \\ \end{gathered} \right.$$

53 $${\text{v}}_{c} (\infty ,t) = \left\{ \begin{gathered} \, 0\,\,\,\,\,\,\,\,\,\,\,\,\,\,\,\,\,\,\,\,\,\,\,\,\,\,\,\,\,\,\,\,\,\,\,\,\,\,\,\,\,\,\,\,\,\,\,\,\,\,\,\,\,\,\,\,\,\,\,\,\,\,\,\,\,\,\,\,\,\,\,\,\,\,\,\,\,\,\,\,\,\,\,\, \, \,if\,\,\varepsilon = 0 \hfill \\ \frac{{M\omega \sin^{2} (\wp )}}{{\Lambda^{2} + \omega^{2} }}e^{ - \Lambda t} + \frac{{M\sin^{2} (\wp )}}{{\sqrt {\Lambda^{2} + \omega^{2} } }}\sin (\omega t - \theta )\,\,\,\,\,\,\,\,\,if\,\,\,\varepsilon = 1, \hfill \\ \end{gathered} \right.$$

are in good compatibility with the Eqs. (50) and (51). The Eqs. (52) and (53) also invole the transient elements of the fluid velocity.

## Results validation

To validate the current acquried results, for $f(t) = H(t)(1 - ae^{ - bt} )$ along with taking $\lambda_{1} = 0,K = 0,\upsilon = 0,\wp = \frac{\pi }{2}$ and $\lambda_{2} = 0$ in Eqs. (34)–(36), then recovered the same solution expressions as Shah et al.[^25^, Eqs. (37), (38) and (39)] obtained for Newtonian fluid case. Moreover, when we are taking $g(t) = H(t)$ together with $\lambda_{1} = 0,\upsilon = 0,\wp = \frac{\pi }{2}$ and $\lambda_{2} = 0$ in relation (34), the corresponding achieved results are same that investigated by Narahari and Debnath[^24^, Eq. (11-a) taking $a_{o} = 0$] and also Tokis[^40^, Eqs. (12)] for thecase without considering the effects of porous, thermal and concentration distribution. Further, it is remarkable to mention that we get the same expression for differnt components of velocity like momentum, thermal, concentration and relations for various cases discussed in section "Different cases relating the motion of the plate", by taking $\lambda_{2} = 0$ and $\Lambda = H$ as A.A. Zafar et al.^39^.

Again, for $f(t) = H(t)(1 - ae^{ - bt} )$ and $g(t) = \cos (\omega t)\,H(t)\,{\text{or}}\,\,\sin (\omega t)H(t)$ along with taking $\lambda_{1} = 0,K = 0,\upsilon = 0,\wp = \frac{\pi }{2}$ and $\lambda_{2} = 0$ in Eq. (34), then recovered the corresponding equations are obtained for viscous fluid case^25^, as follow

$${\text{v}}_{cm} (y,t) = \frac{y}{2\sqrt \pi }\int\limits_{0}^{t} {\frac{{\cos \left[ {\omega (t - s)} \right]}}{s\sqrt s }\exp \left( { - \frac{{y^{2} }}{4s} - \Lambda s} \right)ds + \varepsilon M\int\limits_{0}^{t} {\cos \left[ {\omega (t - s)} \right]e^{ - \Lambda s} erf\left( {\frac{y}{2\sqrt s }} \right)ds} } ,$$

$${\text{v}}_{sm} (y,t) = \frac{y}{2\sqrt \pi }\int\limits_{0}^{t} {\frac{{\sin \left[ {\omega (t - s)} \right]}}{s\sqrt s }\exp \left( { - \frac{{y^{2} }}{4s} - \Lambda s} \right)ds + \varepsilon M\int\limits_{0}^{t} {\sin \left[ {\omega (t - s)} \right]e^{ - \Lambda s} erf\left( {\frac{y}{2\sqrt s }} \right)ds.} }$$

## Results explanation with discussion

The work in the present article examines the analytical solutions of the magneto-free convective flow of Oldroyd-B fluid model that flow through porous inclined plate, saturated in porous media. The problem is formulated and represented in non-dimensional form with suitable new non-dimensional variables, as the free convection for general motions and oscillating movement over an inclined plate which lies in the porous material. For seeking exactsolution expressions in terms of G-function, for Oldroyd-B fluid velocity, concentration and temperature distribution, Laplace integral transformation method is used to solve the presented fluid model. For physical significance of various parameters involved in the problem and exploring the accomplished analytically solutions, various cases for theoretical interest having applications in engineering field are considered, and also parleyed some established results as limiting cases that has been existed in the literature. Results are demonstrated graphically, inorder to keenly analyse the effects of fluidic parameters $$N,$$$$Sc,$$$$R_{C}$$, $$\Pr_{eff}$$, $$\lambda_{1}$$, $$\lambda_{2}$$ and variables especially for the inclined plate (with the vertical) and slanted external magnetic flux on the fluid motion, also discussed for a motion of slowly accelerating plate.

**Figure 2:**
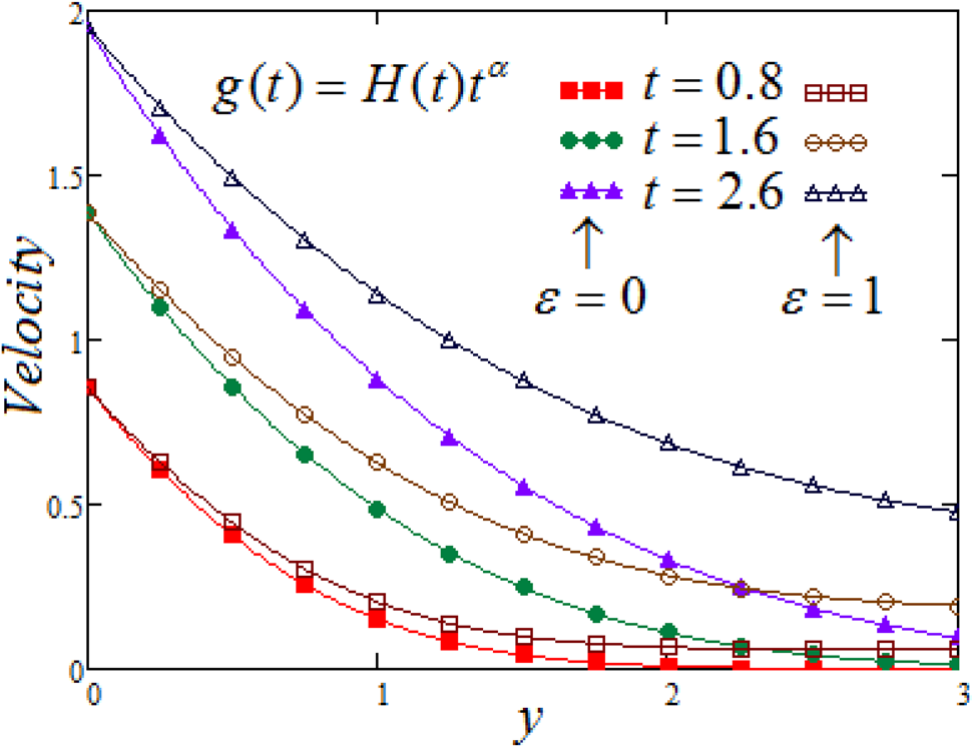
Representation of fluid velocity $${\text{v}}(y,t)$$ against $$y$$ for distinct values of $$t$$ with $$\Pr_{eff} = 4.5,$$$$Sc = 0.8,$$$$\lambda_{1} = 0.7,$$ $$\wp = \tfrac{\pi }{6},\lambda_{2} = 0.3,\upsilon = \tfrac{\pi }{6},K = 1,$$ $$\alpha = 0.7,$$ $$N = 2.5,$$ $$R_{C} = 0.7$$.

Figure 2 is delineated to demonstrate the effect of time on velocity profile ${\text{v}} (y,t)$ in contrast to $y$ at varying values of time. It is perceived that, the velocities elevated for MFFRP case as compare to MFFRF, other than that as it is noted from the figure when y increase asymptotically then corresponding velocity curves declined. Furthermore, it is observed from the figure that, when y tends to infinity then corresponding value of the velocity is non-zero for MFFRP case.

**Figure 3:**
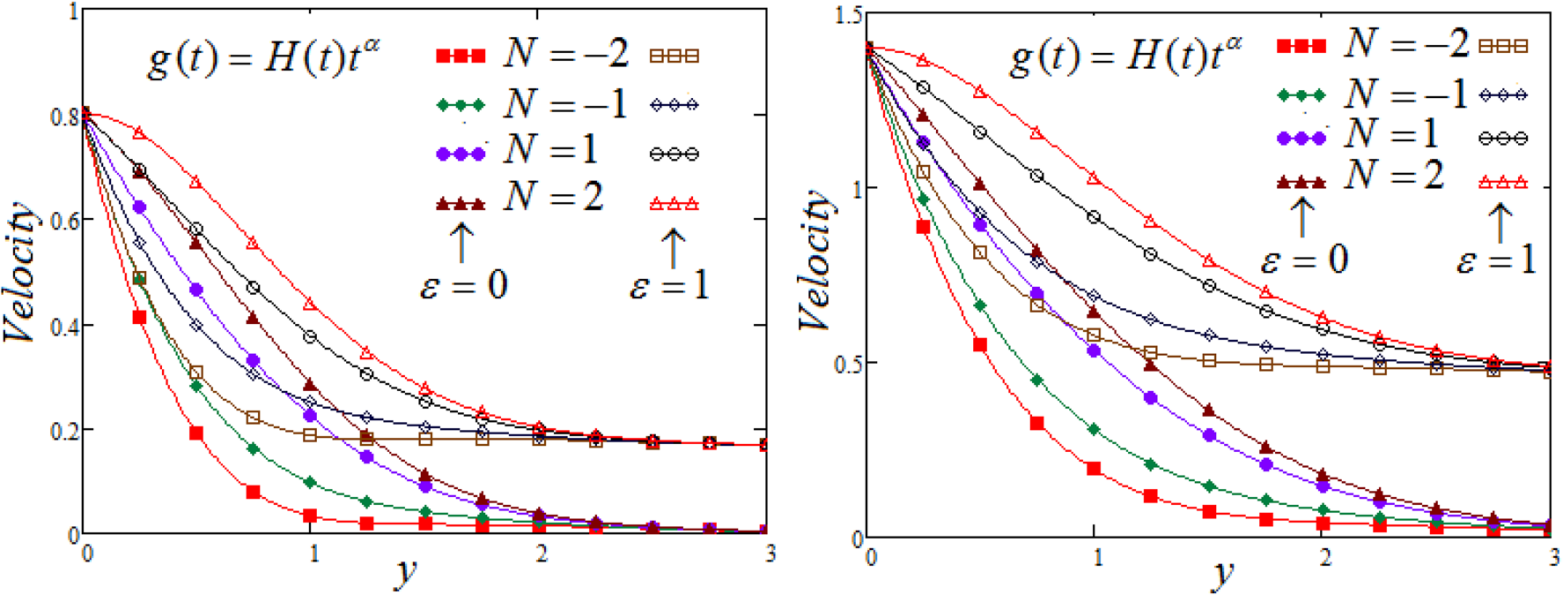
Representation of fluid velocity $${\text{v}}(y,t)$$ against $$y$$ for distinct values of $$N$$ with $$\Pr_{eff} = 4.5,$$$$Sc = 0.8,$$$$\lambda_{1} = 0.7,$$ $$\wp = \tfrac{\pi }{6},\lambda_{2} = 0.3,\upsilon = \tfrac{\pi }{6},K = 1,$$ $$\alpha = 0.7,$$$$R_{C} = 0.7$$.

Figure 3 portrays the influence of N on fluid velocity at two different time, for $t = 0.8,1.4$_._for both cases aiding $N > 0$ and opposing $N < 0$ flows. For ading flows $N > 0$, the thermal buoyancy force to viscous force, as a result, with an increasing in $N.$ cause a remarkable increasing impact on the fluid velocity, have appeared due to boost in the value of $N.$ For opposing flows $N < 0$, then thermal buoyancy force have been opposed by species diffusion that cause to resists the fluid flow, and observed the reversal effect on velocity profile.

**Figure 4:**
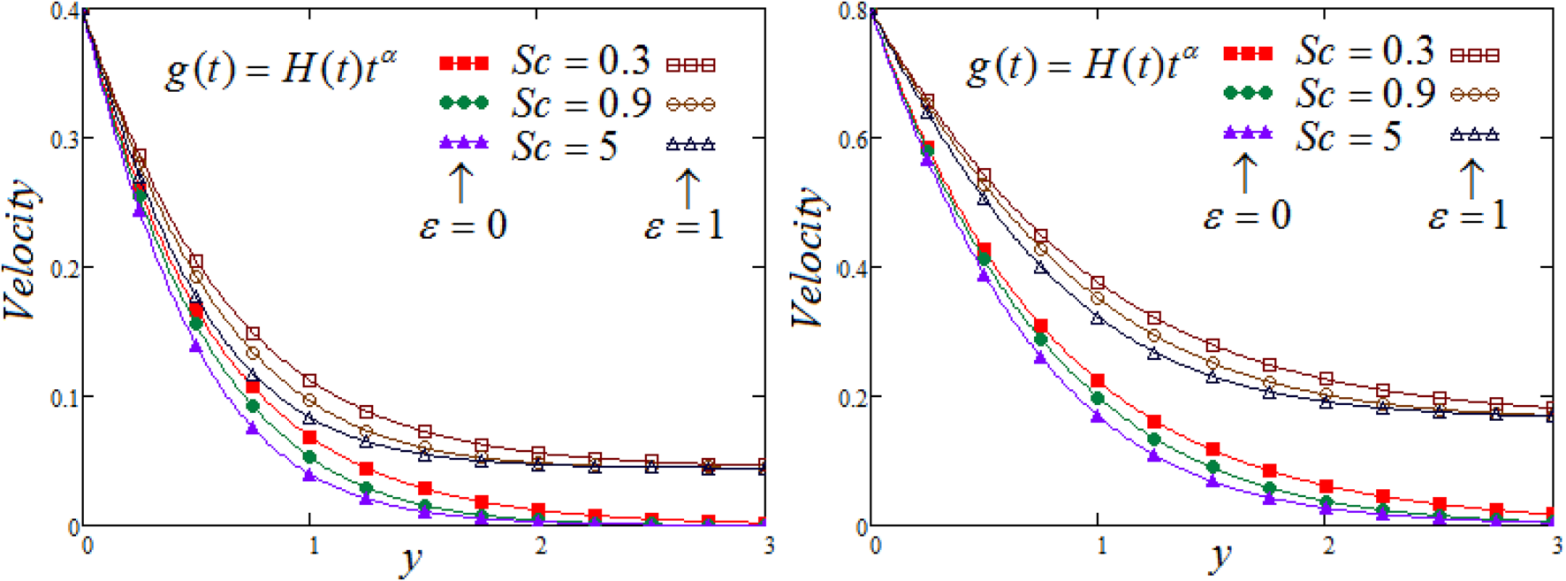
Representation of fluid velocity $${\text{v}}(y,t)$$ against $$y$$ for distinct values of $$Sc$$ with $$N = 2.5,$$$$\Pr_{eff} = 4.5,$$$$\lambda_{1} = 0.7,$$ $$\wp = \tfrac{\pi }{6},\lambda_{2} = 0.3,\upsilon = \tfrac{\pi }{6},K = 1$$,$$\alpha = 0.7,$$$$R_{C} = 0.7$$.

Figure 4 the behaviour of Sc on fluid velocity curve is depicted, against y, for different values of Sc, at two different values of time. It is noticed that a decreasing effect on concentration in the boundary layer when the values of the Schmidt number enlarged. Physically, the relative influence of of momentum diffusivity to species diffusivity is the definition of Schmidt number Sc. It is noticed that, momentum diffusivity is quicker than species diffusivity when Sc is greater than one (Sc > 1), but it is reverse when Sc is less than one (Sc < 1), and in case of (Sc = 1), both species and momentum boundary layers have magnitude of thesame order.

**Figure 5:**
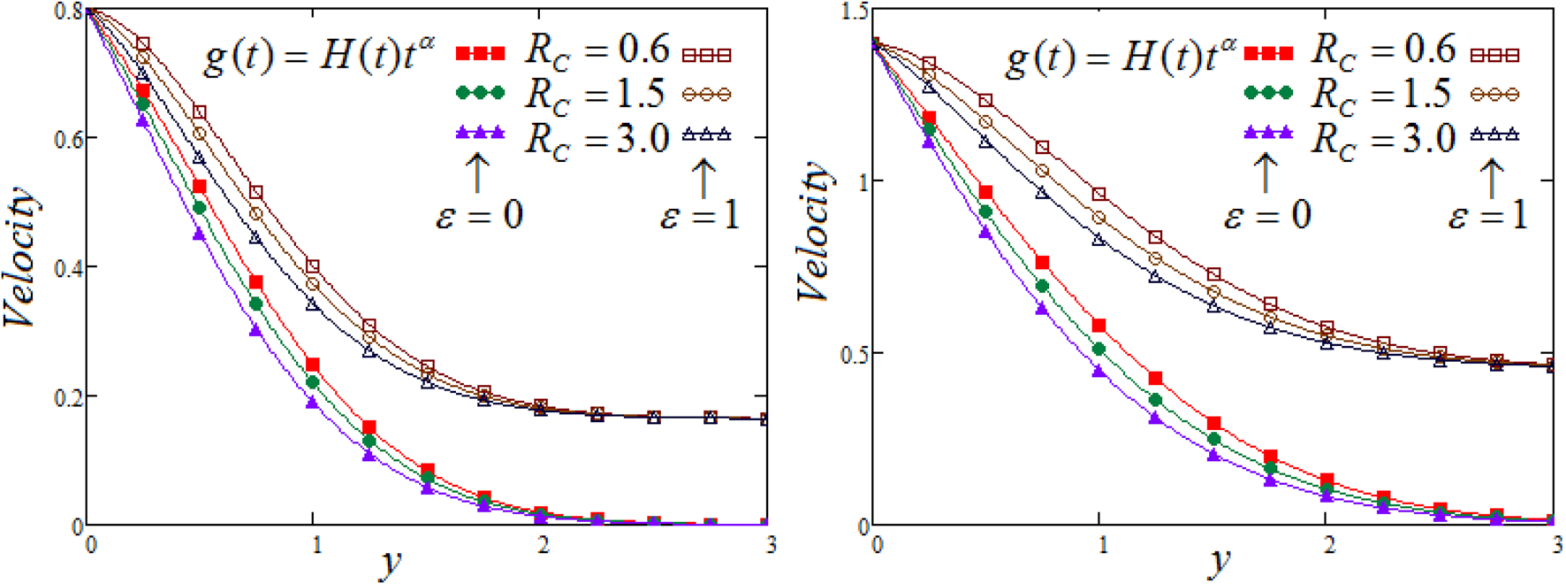
Representation of fluid velocity ${\text{v}}(y,t)$ against $y$ for distinct values of $Rc$ with $N = 2.5,$ $\Pr_{eff} = 4.5,$ $Sc = 0.8,$ $\lambda_{1} = 0.7,$ $\wp = \tfrac{\pi }{6},\lambda_{2} = 0.3,\upsilon = \tfrac{\pi }{6},K = 1$,$\alpha = 0.7$.

Figure 5 illustrates the behavior of $Rc$ (the chemical reaction) on a fluids velocity, at two different values of time. It is realized that the velocity is in a decreases corresponding to the increase in the values of chemical reaction parameter. Also, when chemical reaction parameter is elevated then the fluid concentration is suppressed and throughout the fluid motion process decline in the concentration distribution noticed ultimately the effects of the concentration buoyancy decreases and hence, fluid velocity also decreases.

**Figure 6:**
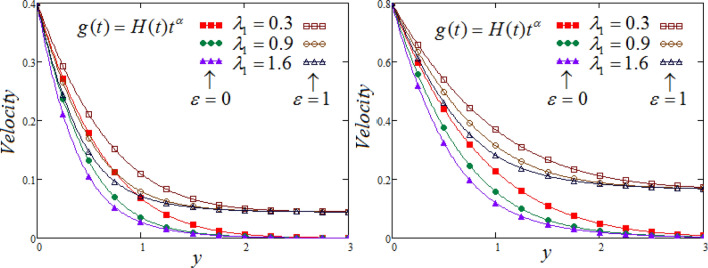
Representation of fluid velocity $${\text{v}}(y,t)$$ against $$y$$ for distinct values of $$\lambda_{1}$$ with $$N = 2.5,$$$$\Pr_{eff} = 4.5,$$$$R_{C} = 0.7$$ $$\wp = \tfrac{\pi }{6},\lambda_{2} = 0.3,\upsilon = \tfrac{\pi }{6},K = 1$$,$$\alpha = 0.7$$$$Sc = 0.8$$.

**Figure 7:**
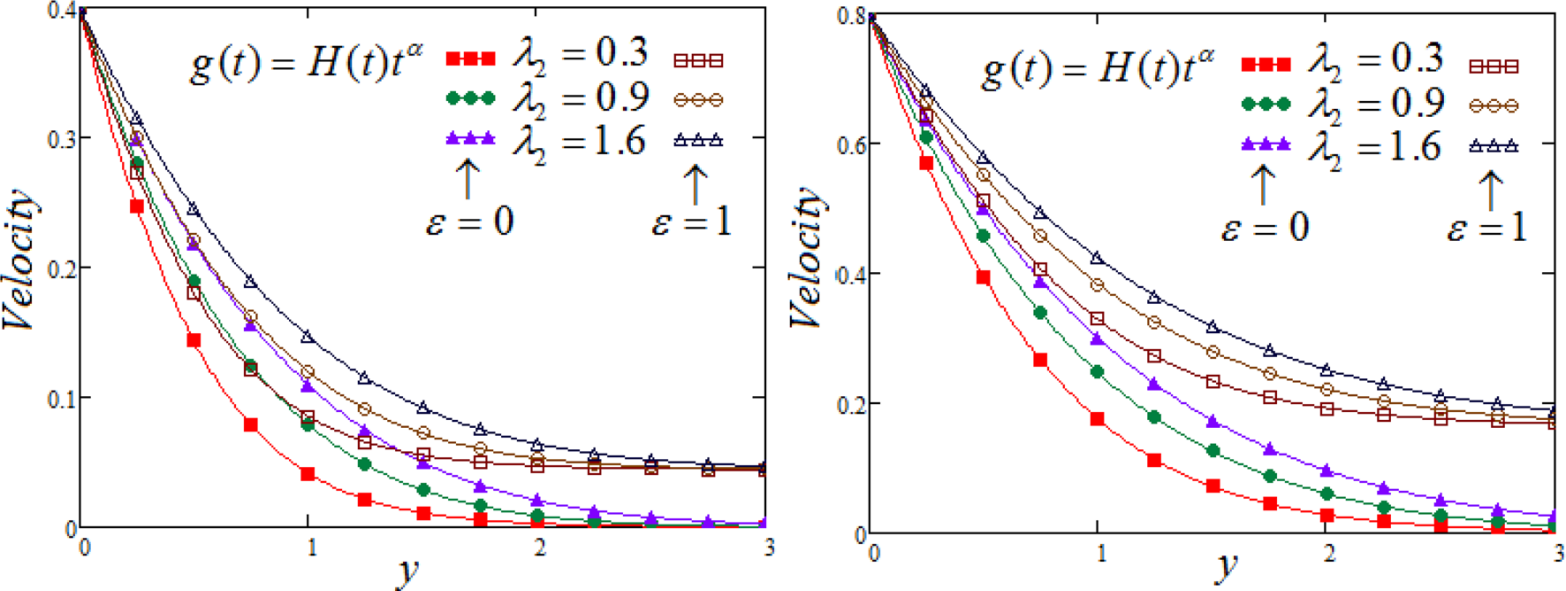
Representation of fluid velocity $${\text{v}}(y,t)$$ against $$y$$ for distinct values of $$\lambda_{2}$$ with $$R_{C} = 0.7,$$$$N = 2.5,$$$$\Pr_{eff} = 4.5,$$ $$\wp = \tfrac{\pi }{6},\lambda_{1} = 0.7,\upsilon = \tfrac{\pi }{6},K = 1$$,$$\alpha = 0.7$$$$Sc = 0.8$$.

Figures 6 and 7 delineated to demonstrates the effect of distinct values of $\lambda_{1}$ and $\lambda_{2}$ on fluid velocity corresponding to y, for different values of $\lambda_{1}$ and $\lambda_{2}$, at two different valuesof time, cosidered both cases such as MFFRP and MFFRF. It is established that the decline in velocity graph due to rise in $\lambda_{1}$ (relaxation time), but opposite trend obseved for $\lambda_{2}$ (retardation time).

**Figure 8:**
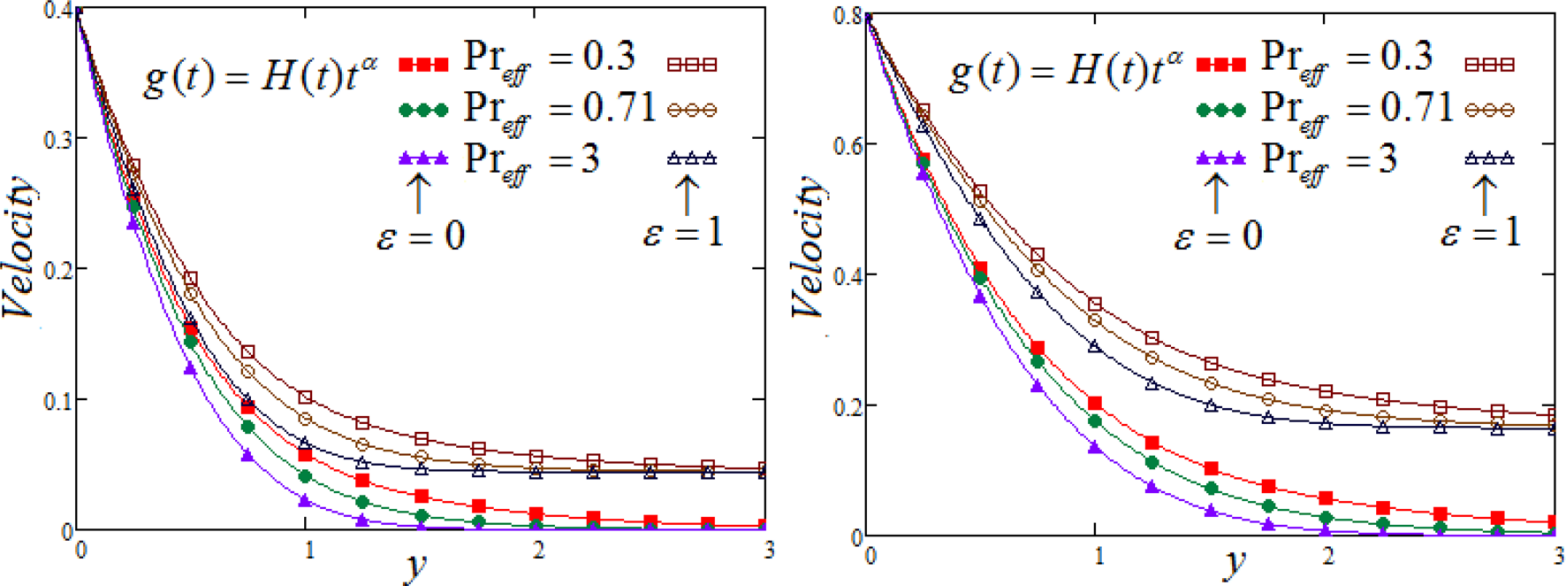
Representation of fluid velocity $${\text{v}}(y,t)$$ against $$y$$ for distinct values of $$\Pr_{eff}$$ with $$N = 2.5,$$$$\lambda_{1} = 0.7,$$ $$\wp = \tfrac{\pi }{6},\lambda_{2} = 0.3,\upsilon = \tfrac{\pi }{6},K = 1$$,$$\alpha = 0.7$$,$$R_{C} = 0.7,$$$$Sc = 0.8$$.

Figure 8 exhibits the effect of Prandtl number $\Pr_{eff}$ on fluid velocity corresponding to y, for different values of $\Pr_{eff}$, at two different values of time. It is notice able that a decreasing effect on velocity in the boundary layer when the values of the Prandtl number enlarged. Physically, an increasing the values of Prandtl number that causes to an increases the fluid viscosity, because of this, fluid becomes thicker due to viscosity increased, as a consequence, fluid velocity decreased.

**Figure 9:**
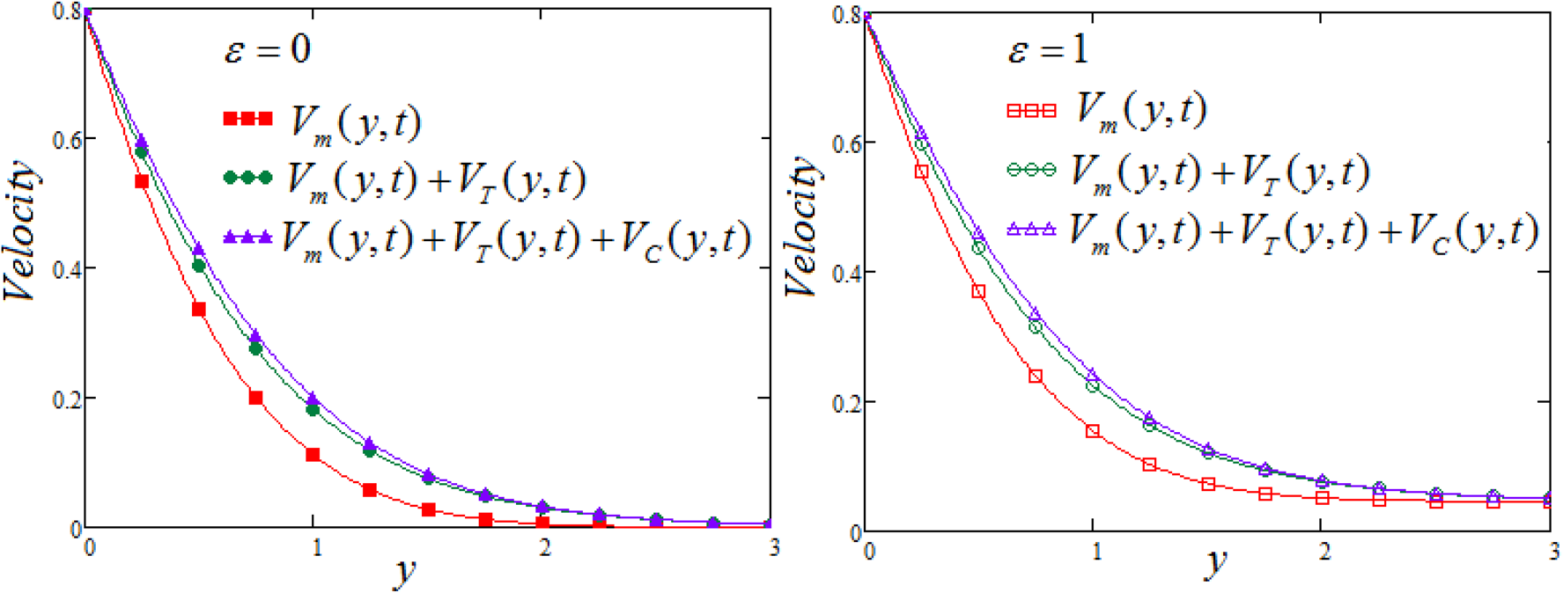
Representation of fluid velocity components against $$y$$ at $$t = 0.8$$ with $$\Pr_{eff} = 4.5,$$$$\lambda_{1} = 0.7,$$ $$\wp = \tfrac{\pi }{6},\lambda_{2} = 0.3,\upsilon = \tfrac{\pi }{6},K = 1,$$ $$R_{C} = 0.7,$$$$Sc = 0.8$$ and $$N = 2.5$$.

Finally, the fluid velocity components (concentration, thermal and mechanical) that contribute in the movement of the fluid, are displayed in Fig. 9, cosidered both cases such as MFFRF and MFFRP. These graphs depict the involvement and significance of each component, which cannot be negligible. It is noticed that, in all figures, the following values have been used $$a = 0.70,\,\,b = 0.10,$$$$M = 0.6.$$, along with system parameters, in all the Figs. 2, 3, 4, 5, 6, 7, 8 and 9.

## Conclusions

In this article, the analytical solutions of the magneto-free convective flow of Oldroyd-B fluid model that flow through porous inclined plate, saturated in porous media. The problem is formulated and represented in non-dimensional form with suitable new non-dimensional variables, as the freeconvection for general motions and oscillating movement over an inclined plate which lies in the porous material. For seeking exact solution expressions in terms of G-function and Mittag–Leffler function, for Oldroyd-B fluid velocity, concentration and temperature distribution, Laplace integral transformation method is used to solve the presented fluid model. For physical significance of various parameters involved in the problem and exploring the accomplished analytically solutions, various cases for theoretical interest having applications in engineering field are considered, and also parleyed some established results as limiting cases that has been existed in the literature. Results are demonstrated graphically, inorder to examine the effects ofsystem parameters for the inclined plate (with the vertical) and slanted external magnetic flux on the fluid motion, also discussed for a motion of slowly accelerating plate. Some main results are concluded as follow:

- It is noticed that the value of fluid velocity does not become zero when it is far away from the plate in case of MFFRP.
- MFFRP (magnetic field is fixed relative to the plate), the velocity graphs are significantly larger than MFFRF(magnetic field is fixed relative to fluid).
- An increase in the values of relaxation time causes to decline velocity, but reverse impact noticed in case of retardation time.
- The increasing variation of buoyancy forces ratio parameter $N$ causes to accumulate the fluid velocity in either MFFRF or MFFRP cases.
- It is depicted that decay in fluid velocity is observed against the raising values of parameters $\Pr_{eff}$,Sc and Rc for both cases MFFRP and MFFRF.
- It is observed that the three velocity components which play significant role and contributions of these components cannot be ignored.

## Data Availability

The data used in current study, available within the article that support the findings of the present research work.
